# 64 Community Level Disadvantage Negatively Impacts Return to Work After Burn Injury

**DOI:** 10.1093/jbcr/irac012.067

**Published:** 2022-03-23

**Authors:** Schachia Murphy, Kara McMullen, Gretchen J Carrougher, Stephanie A Mason, Damien W Carter, Barclay T Stewart, Callie M Thompson, Oscar E Suman-Vejas

**Affiliations:** Maine Medical Center, Portland, Maine; University of Washington, Portland, Oregon; Department of Surgery, The University of Washington, Seattle, Washington; Sunnybrook Health Sciences Centre, University of Toronto, Toronto, Ontario; Maine Medical Center, Portland, Maine; University of Washington, Seattle, Washington; University of Utah Health, Salt Lake City, Utah; U of Texas Medical Branch, Galveston, Texas

## Abstract

**Introduction:**

The loss of income from injury, additional health care expenses, and inability to return work can lead to unsatisfactory outcomes. Community level disadvantage (e.g., low high school completion, low home ownership, low income) are more common among minority groups. We hypothesized community level disadvantage would negatively impact the ability to return to work after burn injury. This could serve to identify patients who need focused social, vocational, and financial support during rehabilitation.

**Methods:**

Data from adult participants in a large multicenter database from 1998-2021 were linked by zip-code to three multi-domain community level-indices: i) Distressed Communities Index, ii) Social Vulnerabilities Index (SVI), iii) Social Deprivation Index (SDI). Cohort characteristics, the distribution of each index within cohort, and days to return to work were described. Fit and strength of association between the indices and return to work was assessed with multi-level logistic regression models. A non-responder analysis examining demographic and clinical differences between was performed using Chi-square tests and Wilcoxon rank sum tests to understand potential bias in the findings.

**Results:**

A total of 1960 participants provided both zip code and employment data 6 months after injury. 75% of participants were male. Mean age was 39. Race/Ethnicity Data: 81.4% identified as White, 11% Black, and 7% as “other” race; 84% of the participants as non-Hispanic or Latino. Median burn size was 20% TBSA (IQR 0.1-95.0), and length of hospitalization was 30 days (IQR 0-379). Of the community indices tested, both DCI and SVI were associated with return to work with DCI having the strongest association with return to work after injury, irrespective of indices. However, when DCI and SVI were included in the model to represent community disadvantage, the impact of race on return to work was less. Participants who did not provide employment information were younger, sustained larger burn sizes, and had longer LOS compared to those who did.

**Conclusions:**

DCI and SVI are associated with return to work after burn injury and can be used to focus limited social, vocational, and financial services. Minoritized participants were less likely to return to work but they live in communities with greater disadvantage (e.g. fewer employment opportunities), which highlights the public health impacts of structural racism.

